# EuroQol-5 dimensions utility gain according to British and Swedish preference sets in rheumatoid arthritis treated with abatacept, rituximab, tocilizumab, or tumour necrosis factor inhibitors: a prospective cohort study from southern Sweden

**DOI:** 10.1186/s13075-016-0950-0

**Published:** 2016-02-19

**Authors:** Anders Gülfe, Johan K. Wallman, Lars Erik Kristensen

**Affiliations:** Department of Clinical Sciences Lund, Rheumatology Section, Lund University, 221 84 Lund, Sweden; Department of Rheumatology, Skåne University Hospital, 221 85 Lund, Sweden; The Parker Institute, Department of Rheumatology, Copenhagen University Hospital, Bispebjerg and Frederiksberg, 2000 Copenhagen, Denmark

**Keywords:** Rheumatoid arthritis, Quality of life, Biological treatment, Observational study

## Abstract

**Background:**

The development of EuroQol-5 dimensions (EQ-5D) utility over time in rheumatoid arthritis (RA) patients, treated with biologics other than tumour necrosis factor inhibitors (TNFi), based on the standard British (UK) and the new Swedish (SE) EQ-5D preference sets, has not been previously described.

**Methods:**

Demographics, core set data, EQ-5D utility, and treatment characteristics for patients with established RA, receiving biologics in southern Sweden from January 2006 to March 2014, were retrieved from observational databases. Theoretical, UK, and experience-based, SE, EQ-5D mean utilities were plotted over time.

**Results:**

Data regarding 2418 treatment courses with abatacept (ABA, n = 100), rituximab (RTX, n = 230), tocilizumab (TOC, n = 121), or TNFi (n = 1967) were included in the analysis. Patients starting TNFi treatment, as expected, had shorter disease duration and less previous biologics. Baseline utilities of patients commencing ABA and TOC, but not RTX, were also lower than in the TNFi group. Following treatment initiation, rapid utility improvements were seen with all therapies, reaching plateaus after approximately 1.5 months, and then remaining fairly stable throughout follow-up in patients adhering to therapy. SE utilities were consistently higher than UK, with baseline values at around 0.7 leaving little room for improvement.

**Conclusions:**

ABA, RTX, TOC, and TNFi treatments were all associated with favourable EQ-5D utility developments in RA patients adhering to therapy. The compression of the experience-based SE preference set towards higher utilities may compromise its ability to detect between-group differences in quality-adjusted life-years, thus making cost-effectiveness harder to demonstrate in cost-utility analyses applying this preference set, rather than the standard UK.

## Background

Cost-utility analyses (CUA) of novel, costly treatments – e.g. biologics for rheumatoid arthritis (RA) – are increasingly used to aid resource allocation, and are frequently referred to by stakeholders, payers, authors of treatment guidelines and health authorities [1–3]. CUAs are based on estimates of incremental costs in relation to incremental accumulation of quality-adjusted life-years (QALYs), typically comparing a recent intervention to standard treatment or placebo. QALYs, in turn, are calculated as the area under the utility curve plotted against time, with utility being a measure of health-related quality of life (HRQoL), anchored at 0 (death) and 1 (perfect health). Utility is usually estimated by means of generic, preference-based HRQoL instruments, such as the EuroQol-5 dimensions (EQ-5D) descriptive system, consisting of five questions on mobility, self-care, usual activities, pain/discomfort and anxiety/depression, where the respondent is asked to rate his/her problems at one of three levels (no problem; some problem; unable to do) [4]. The resulting response constitutes one of 243 possible health states, each of which have been assigned a specific utility value by means of a valuation process in a reference population. The reference persons are asked to estimate either their own health (experience-based valuation) or health states described to them (hypothetical valuation) by means of a direct HRQoL instrument, such as time trade-off (TTO), standard gamble (SG) or visual analogue scale (VAS). An algorithm, describing the translation of each health state into a utility value, will then be computed. The resulting weights (preference set) will thus depend on the algorithm and on the properties of the reference population, e.g. demographics, sociocultural factors, health and a variety of methodological issues, including the choice between hypothetical or experience-based valuation [5].

The first and most widely used EQ-5D preference set was derived by hypothetical valuation in a British (UK) reference population [6], but there are several other national preference sets available, based variously on hypothetical or experience-based, TTO or VAS valuations, each giving rise to different utilities for the same health states [7, 8]. Whereas the use of such national preference sets may indeed help reflect the values of the population in which a CUA is conducted, the variability of results complicates the comparison of studies. Moreover, national health authorities may express support for certain methodologies, influencing the development in their respective countries – the stance of the Swedish Dental and Pharmaceutical Benefits Agency (Tandvårds- och Läkemedelsförmånsverket) e.g. being reflected in the use of experience-based rather than hypothetical valuations in the creation of the recently published Swedish (SE) EQ-5D preference set [1, 9].

We have previously demonstrated that EQ-5D utility gain in tumour necrosis factor inhibitor (TNFi) treatment of established RA is rapid, and that utility remains stable in those remaining on therapy for up to 7 years [10]. The development of utility over time in RA patients treated with other biologics is, however, less well known. Thus, the present study aims to (i) describe the utility development during 18 months in established RA patients starting treatment with abatacept (ABA), rituximab (RTX), tocilizumab (TOC) or, for comparison, TNFi; and (ii) to compare the performance of the hypothetical UK and the experience-based SE EQ-5D preference sets in this dataset.

## Methods

Demographics, core set data, type and date (start; stop) of treatment and EQ-5D health states at baseline, 2 and 6 weeks, 3, 9, 12 and 18 months were retrieved from observational databases in southern Sweden for patients treated with ABA, RTX, TOC, or TNFi in clinical practice. The study period constituted January 2006 to March 2014. Up to 2012, observational data was routinely entered into the South Swedish Arthritis Treatment Group (SSATG) register [11], whereas later the Swedish Rheumatology Quality Register (SRQ) was used [12]. Diagnoses were as judged by the treating rheumatologists, which – in a subset of patients – have previously been demonstrated to agree with the 1987 American College of Rheumatology (ACR) criteria in 98 % of cases [13, 14]. Mean (95 % CI) EQ-5D utilities for the time points stated were calculated applying the UK and SE preference sets and plotted against time. Treatment courses lacking baseline EQ-5D data were excluded, and individual patients could contribute data for more than one treatment course due to switching between therapies. The Kaplan-Meier method was used to estimate the number of patients remaining on therapy at each time point. Analysis of covariance was used to compare EQ-5D UK/SE utility change from baseline to 18 months (18-month values imputed by last observation carried forward, LOCF) between the four treatment modalities, adjusting for sex, disease duration, previous number of biologic treatments, and baseline EQ-5D utility (according to the respective preference sets), health assessment questionnaire (HAQ) and 28-joint disease activity (DAS28) scores. Correlations between the 18-month changes in EQ-5D UK/SE utility and HAQ scores (again based on LOCF imputed data) for the respective therapies were also assessed by Spearman’s rho.

The study was approved by the Regional Ethical Review Board in Lund, number 2013/208. Oral informed consent was obtained at first inclusion of data and documented in the database. Due to the quality control and safety surveillance character of the registers, no written consent was required.

## Results

During the study period, 2418 treatment courses of ABA (n = 100), RTX (n = 230), TOC (n = 121) or TNFi (n = 1967), with EQ-5D data available at baseline, were initiated among 1757 patients. Baseline characteristics of subjects commencing the various treatments are given in Table 1. Compared to the other treatments, as expected, patients initiating TNFi therapy had lower point estimate means for number of previous biologics, disease duration, HAQ and DAS28 scores. The point estimate number of previous biologics was also lower among patients commencing RTX, as compared to the ABA and TOC groups.

**Table 1:** Baseline characteristics by therapy

	Abatacept	Rituximab	Tocilizumab	TNFi
N	100	230	121	1967
Age, years	59.0 (12.1)	60.2 (12.3)	57.9 (13.5)	56.6 (13.6)
Female, n (%)	82 (80.4)	166 (72.2)	98 (80.3)	1520 (77.3)
Disease duration, years	14.1 (9.6)	15.6 (11.0)	14.1 (9.6)	11.6 (10.8)
Baseline HAQ	1.46 (0.61)	1.35 (0.67)	1.43 (0.64)	1.18 (0.64)
Baseline DAS28	5.81 (1.30)	5.30 (1.48)	5.71 (1.32)	5.09 (1.32)
Number of previous biologic courses	2.5 (1.5)	1.8 (1.4)	2.3 (1.4)	0.6 (1.0)
Number of ongoing DMARDs^a^	0.7 (1.0)	0.8 (0.6)	0.7 (0.6)	0.9 (0.6)
Ongoing steroids, yes, n (%)	66 (64.7)	156 (67.8)	82 (67.2)	1164 (59.2)

Values are mean (SD) unless stated otherwise

*TNFi* tumour necrosis factor inhibitors, *HAQ* health assessment questionnaire, *DAS28* 28-joint disease activity score, *DMARD* disease-modifying anti-rheumatic drug

^a^Excluding ongoing biologics

**Fig. 1:**
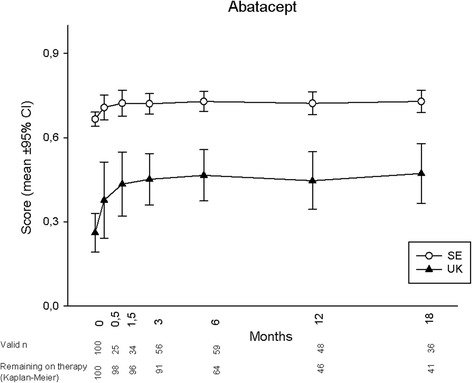
EuroQoL-5 dimensions utility development according to British (UK) and Swedish (SE) preference sets in established rheumatoid arthritis treated with abatacept

**Fig. 2:**
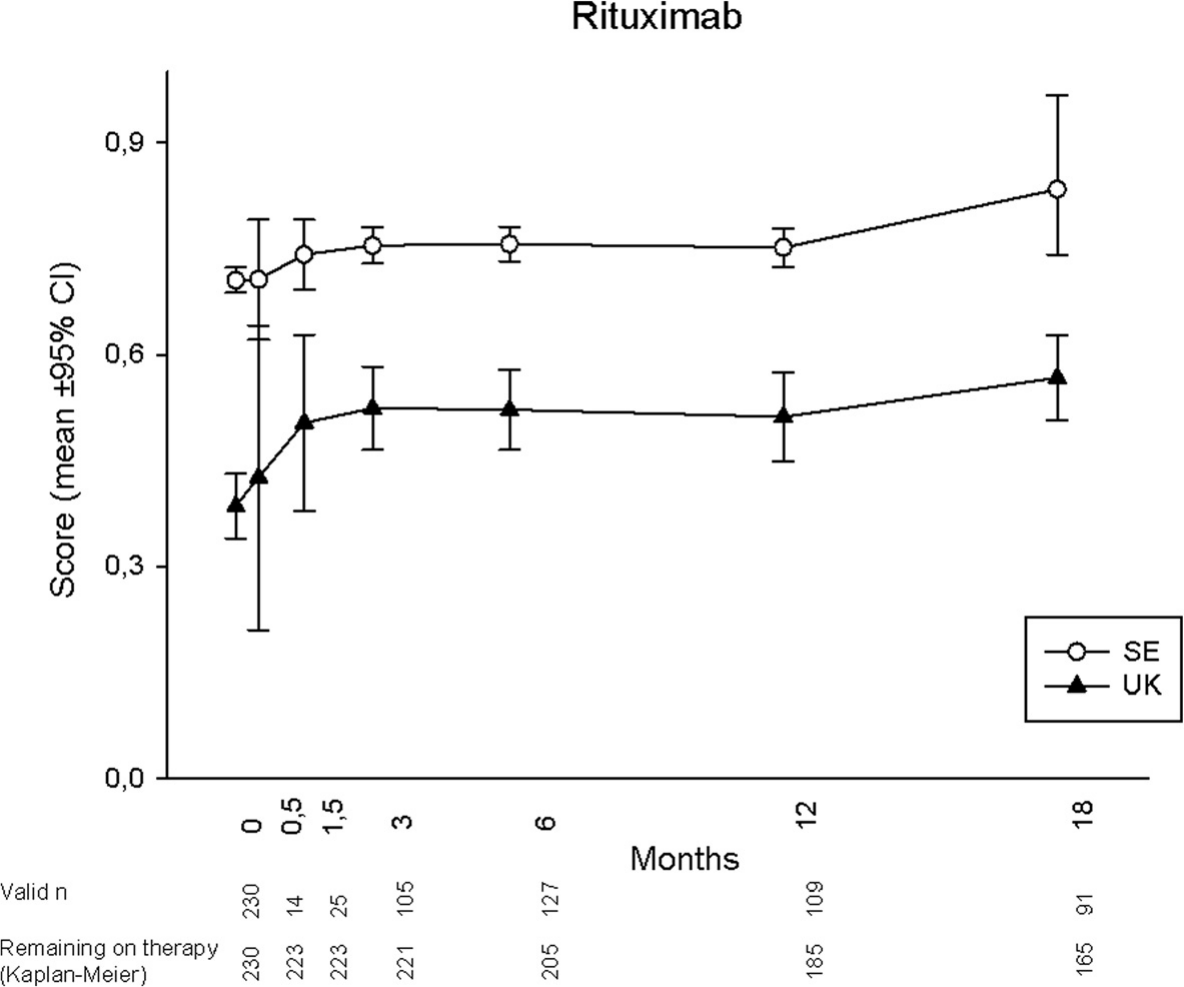
EuroQoL-5 dimensions utility development according to British (UK) and Swedish (SE) preference sets in established rheumatoid arthritis treated with rituximab

**Fig. 3:**
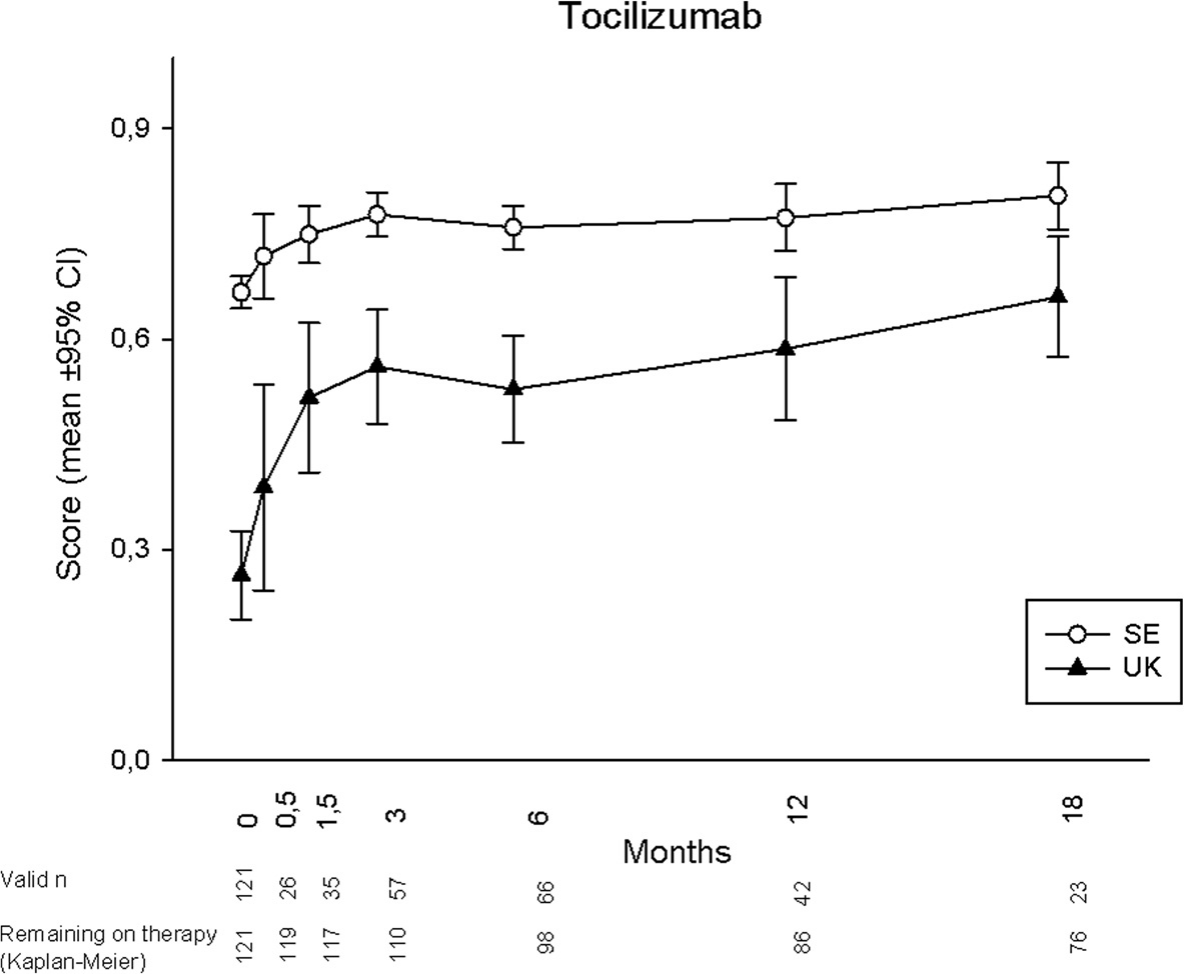
EuroQoL-5 dimensions utility development according to British (UK) and Swedish (SE) preference sets in established rheumatoid arthritis treated with tocilizumab

**Fig. 4:**
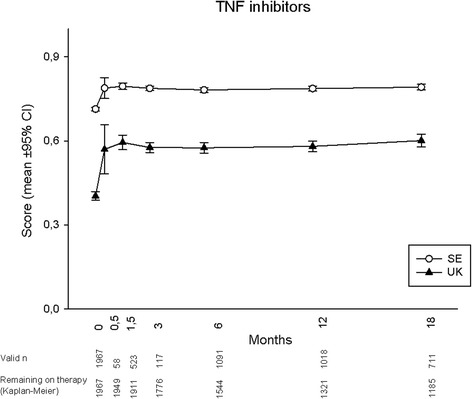
EuroQoL-5 dimensions utility development according to British (UK) and Swedish (SE) preference sets in established rheumatoid arthritis treated with tumour necrosis factor inhibitors

Mean utility development during 18 months for each treatment is displayed in Figs. 1, 2, 3 and 4, according to both UK and SE EQ-5D preference sets. At baseline, mean (95 % CI) UK utilities were 0.26 (0.19, 0.33), 0.39 (0.34, 0.43), 0.26 (0.20, 0.33) and 0.40 (0.39, 0.42) for patients starting ABA, RTX, TOC and TNFi, respectively, while the corresponding SE values were 0.67 (0.64, 0.69), 0.70 (0.69, 0.72), 0.67 (0.64, 0.69) and 0.71 (0.71, 0.72). Patients commencing ABA or TOC therapy thus started from lower mean utility levels, whereas the RTX-treated subjects started from an average utility level similar to those initiating TNFi. Following treatment initiation, rapid utility improvements were seen with all therapies, reaching plateaus after approximately 1.5 months, and then remaining fairly stable throughout follow-up in patients adhering to therapy. Numerically, a somewhat smaller UK utility gain was observed among patients treated with RTX, as compared to all other treatments. When adjusting baseline characteristics, however, the analysis of covariance models did not show any significant difference in EQ-5D UK or SE utility change over 18 months between any of the treatment modalities (*p* >0.1 for all comparisons).

Improvement in utilities was accompanied by improvement in HAQ disability. Spearman correlation between EQ-5D UK and HAQ improvements from baseline to 18 months was 0.53, -0.51, -0.59 and -0.48 for ABA, RTX, TOC and TNFi, respectively. For EQ-5D SE, the corresponding values were -0.52, -0.47, -0.67 and -0.52.

Regarding the choice of EQ-5D preference set, the experience-based SE utilities were consistently higher than the hypothetically derived UK, with baseline values at around 0.7 leaving little room for improvement. Consequently, SE utility gains were numerically much smaller than UK for all treatments, and potential differences between the therapies thus less apparent. The overall patterns of utility development with plateaus from around 1.5 months, however, remained similar to the UK findings.

## Discussion

Based on observational data from southern Sweden, the current study demonstrated a rapid improvement of EQ-5D utility in RA patients commencing treatment with ABA, RTX, or TOC, and that this improvement was sustained for at least 18 months in patients adhering to therapy. Data for patients initiating TNFi treatment is also provided for comparison.

Compared to patients starting TNFi or RTX, the lower mean baseline utilities of the ABA and TOC groups are likely explained by more treatment-refractory disease, as signalled by their higher mean numbers of previous biologics. Mean DAS28 and HAQ scores, both known to correlate with EQ-5D utility [15, 16], were also numerically worse in these groups at treatment initiation. In view of the baseline characteristics, the finding that patients commencing RTX had mean baseline utility on par with the TNFi group was, however, more unexpected, and reasons for this remain partly unresolved.

The pattern of relative stability of utilities in patients remaining on therapy, once treatment response had been achieved, was similar for all treatment modalities, and is well in line with previous findings limited to TNFi therapy [10].

Due to the observational setting, with patient groups not being entirely comparable, the numerically lower UK utility gain in the RTX group should be interpreted with caution, and did not differ significantly from those of the other treatments after adjustment for baseline differences. Despite starting from similar mean utility levels, in relation to the TNFi group, the average RTX patient had indeed more long-standing and treatment-refractory disease (Table 1). On the opposite side, the numerically larger mean UK utility gains of the ABA/TOC groups may at least partly be driven by instrument effects, since when starting from gradually worse EQ-5D health states, ever smaller improvements of the questionnaire responses are required to achieve the same UK utility gain [17]. For comparison, in terms of HAQ developments (data not shown), the RTX group more closely resembled those of patients commencing ABA or TOC than TNFi. Finally, in randomized controlled trials, the efficacy of all the studied treatment modalities has been found to be of similar magnitude [18, 19].

Another main point of interest of the current study is the substantial difference observed between the hypothetical UK and the experience-based SE utilities, derived from the same EQ-5D questionnaire answers. Even though some gain in SE utility occurred over time, the compression of the SE preference set – with the worst possible health state, 33333, corresponding to a utility score of 0.34, as compared to -0.59 by use of the UK weights – clearly constrains the magnitude of potential improvement. Differences in QALY accumulation between interventions with varying efficacy may thus also be lower when applying the SE, as opposed to the UK, preference set, potentially affecting the conclusion of CUAs. While disparities also exist between different hypothetical EQ-5D preference sets [8], experience-based valuations are known to result in higher utilities [9], and likely explain the pronounced differences observed between the UK and SE results.

As has already been alluded to, the open, non-randomized nature of observational studies generates methodological limitations. Confounding by indication as well as assessment and performance bias cannot be excluded from this study. Any direct head-to-head drug interpretations should thus be done with care, and need to be confirmed in other studies and different settings. Moreover, we chose to group all anti-TNF remedies together, rather than to perform separate analyses for the various agents. Another limitation is, EQ-5D data was only available from patients remaining on therapy and the presented figures are based on observed data only, thus introducing attrition bias. Rather than describing mean utility developments of all patients starting the various treatments, the current data should be interpreted as reflecting the situation in those adhering to therapy. On the other hand, by only including unimputed data in the figures, they describe what has actually been observed in the study population.

## Conclusions

The present study showed rapid and sustained EQ-5D utility improvements in RA patients commencing and adhering to treatment with ABA, RTX, TOC and TNFi. Moreover, large differences were observed between EQ-5D utilities derived by use of the hypothetical UK or the experience-based SE preference sets, underscoring the important impact the choice of EQ-5D preference set may have on the outcome and interpretation of CUAs applying this instrument.

## Abbreviations

ABA: Abatacept
ACR: American College of Rheumatology
CUA: Cost-utility analysis
DAS28: 28-Joint disease activity score
EQ-5D: EuroQoL-5 dimensions
HAQ: Health assessment questionnaire
HRQoL: Health-related quality of life
QALY: Quality-adjusted life-years
RA: Rheumatoid arthritis
RTX: Rituximab
SE: Swedish
SG: Standard gamble
SRQ: Swedish Rheumatology Quality Register
SSATG: South Sweden Arthritis Treatment Group
TNFi: Tumour necrosis factor inhibitor
TOC: Tocilizumab
TTO: Time trade-off
UK: British
VAS: Visual analogue scale

## References

[1] Tandvårds- och Läkemedelsförmånsverket. http://www.tlv.se. Accessed 6 July 2015.

[2] National Institute for Health and Care Excellence. http://www.nice.org.uk. Accessed 6 July 2015.

[3] Svensk Reumatologisk Förening. http://www.svenskreumatologi.se. Accessed 6 July 2015.

[4] The EuroQol Group (1990). EuroQoL - a new facility for the measurement of health-related quality of life. Health Policy.

[5] Harrison MJ, Bansback NJ, Marra CA, Drummond M, Tugwell PS, Boonen A (2011). Valuing health for clinical and economic decisions: directions relevant for rheumatologists. J Rheumatol.

[6] Harrison MJ, Davies LM, Bansback NJ, Ingram M, Anis AH, Symmons DP (2008). The validity and responsiveness of generic utility measures in rheumatoid arthritis: a review. J Rheumatol.

[7] Nan L, Johnson JA, Shaw JW, Coons SJ (2007). A comparison of EQ-5D index scores derived from the US and UK population-based scoring functions. Med Decis Making.

[8] Karlsson JA, Nilsson JA, Neovius M, Kristensen LE, Gulfe A, Saxne T (2011). National EQ-5D tariffs and quality-adjusted life-year estimation: comparison of UK, US and Danish utilities in south Swedish rheumatoid arthritis patients. Ann Rheum Dis.

[9] Burstrom K, Sun S, Gerdtham UG, Henriksson M, Johannesson M, Levin LA (2014). Swedish experience-based value sets for EQ-5D health states. Qual Life Res.

[10] Gulfe A, Kristensen LE, Saxne T, Jacobsson LT, Petersson IF, Geborek P (2010). Rapid and sustained health utility gain in anti-tumour necrosis factor-treated inflammatory arthritis: observational data during 7 years in southern Sweden. Ann Rheum Dis.

[11] Geborek P, Saxne T. Clinical protocol for monitoring of targeted therapies in rheumatoid arthritis. Rheumatology (Oxford). 2000;39:1159–61. 10.1093/rheumatology/39.10.1159 11035144

[12] Svensk Reumatologis Kvalitetsregister. http://www.srq.nu. Accessed 6 July 2015.

[13] Arnett FC, Edworthy SM, Bloch DA, McShane DJ, Fries JF, Cooper NS (1988). The American Rheumatism Association 1987 revised criteria for the classification of rheumatoid arthritis. Arthritis Rheum.

[14] Geborek P, Crnkic M, Petersson IF, Saxne T (2002). Etanercept, infliximab, and leflunomide in established rheumatoid arthritis: clinical experience using a structured follow up programme in southern Sweden. Ann Rheum Dis.

[15] Hurst NP, Kind P, Ruta D, Hunter M, Stubbings A (1997). Measuring health-related quality of life in rheumatoid arthritis: validity, responsiveness and reliability of EuroQol (EQ-5D). Br J Rheumatol.

[16] Radner H, Smolen JS, Aletaha D (2014). Remission in rheumatoid arthritis: benefit over low disease activity in patient reported outcomes and costs. Arthritis Res Ther.

[17] Dolan P (1997). Modeling valuations for EuroQol health states. Med Care.

[18] Kristensen LE, Christensen R, Bliddal H, Geborek P, Danneskiold-Samsoe B, Saxne T (2007). The number needed to treat for adalimumab, etanercept, and infliximab based on ACR50 response in three randomized controlled trials on established rheumatoid arthritis: a systematic literature review. Scand J Rheumatol.

[19] Kristensen LE, Jakobsen AK, Bartels EM, Geborek P, Bliddal H, Saxne T (2011). The number needed to treat for second-generation biologics when treating established rheumatoid arthritis: a systematic quantitative review of randomized controlled trials. Scand J Rheumatol.

